# Psychoeducation focused on family accommodation: a practical intervention for parents of children and adolescents with obsessive-compulsive disorder

**DOI:** 10.1186/s13052-021-01177-3

**Published:** 2021-11-06

**Authors:** Francesco Demaria, Maria Pontillo, Maria Cristina Tata, Prisca Gargiullo, Francesco Mancini, Stefano Vicari

**Affiliations:** 1 Child and Adolescence Neuropsychiatry Unit, Department of Neuroscience, Children Hospital Bambino Gesù, IRCCS, Viale Ferdinando Baldelli 41, 00146 Rome, Italy; 2 Scuola di Psicoterapia Cognitiva APC-SPC, Viale Castro Pretorio, 116, 00185 Rome, Italy; 3 Department of Life Sciences and Public Health, Catholic University of the Sacred Heart, 00168 Rome, Italy

**Keywords:** Obsessive-compulsive disorder, Children, Adolescents, Cognitive-behavioral therapy, Psychoeducation

## Abstract

Obsessive-compulsive disorder (OCD) is a neuropsychiatric disorder that is frequently diagnosed in children and adolescents. In pediatric OCD, family plays an important role in the development and maintenance of the disease. In this relationship, both genetic and behavioral factors, such as parental modeling and family accommodation, are significant. Parental modeling concerns the daily enactment of dysfunctional behavioral patterns by a parent with OCD, which may influence children. Family accommodation, in contrast, describes the direct participation of parents in their child’s compulsive rituals, by modifying daily routines or by facilitating avoidance of OCD triggers, to decrease the child’s distress and time spent executing compulsions. Approximately 80–90% of the relatives of OCD patients actively participate in patients’ rituals. The literature demonstrates that a high level of family accommodation is associated with OCD symptom severity, reduced response to cognitive-behavioral treatment (CBT), and a higher risk of therapy dropout.

Despite this, no studies have aimed at delineating practical guidance for psychotherapists to support parents in reducing family accommodation.

The main aim of this paper is to propose a psychoeducation intervention focused on cognitive-behavioral strategies to help families to manage their child’s OCD behaviors without enacting dysfunctional family accommodation behaviors in order to support their child’s successful therapy.

## OCD in children and adolescents: clinical presentation

Obsessive-compulsive disorder (OCD) is a neuropsychiatric disorder characterized by obsessive thoughts (i.e., intrusive, repetitive, unwanted thoughts), accompanied by compulsive behaviors or mental acts [5]. The disorder has a worldwide prevalence of 0.25–3.0% [60], while its estimated prevalence among children and adolescents is 1–3% [59]. In this population, untreated OCD symptomatology follows a chronic course [22, 51, 53] and is related to significant impairment in quality of life and social, scholarly, and family functioning [3, 12, 22, 25, 52]. Amongst adults with OCD, 30–50% experience the onset of symptoms prior to the age of 18 years [33]. Early onset of OCD symptomatology occurs before the age of 10 years [34], and is more frequently reported in males. Often, early onset OCD children report a comorbidity with attention deficit hyperactivity disorder (ADHD) [17, 18].

Research suggests that the age of onset could determine differential expressions of clinical OCD symptoms [17, 18, 26]. For example, OCD in childhood and adolescence is predominately associated with the male gender, a higher frequency of aggression/catastrophe obsessions (including fears of catastrophic events, such as the death or illness of one’s self or loved ones), increased hoarding and saving compulsions, and poorer insight, compared with adult OCD patients. Sexual and religious obsessions are more prevalent in OCD adolescents, relative to children or adults with OCD [17]. In particular, male adolescents with OCD report more sexual obsessions, while female adolescent OCD patients describe more hoarding compulsions [27]. In contrast, adult female OCD patients report more contamination and cleaning symptoms [10, 23, 58], while adult male OCD patients report more order and symmetry symptoms, associated with tics [28], as well as hoarding symptoms [48].

Comorbid psychopathological disorders or subclinical symptoms are frequent in OCD children and adolescents [19]. Storch et al. [57] found that 74% of youth with OCD met the criteria for at least one comorbid diagnosis, and children with at least one comorbid diagnosis had a lower treatment response and remission rate with CBT compared to those without a comorbid diagnosis. According to Peris et al. [40], 50% of a sample of children and adolescents with OCD (*N* = 322) reported a comorbid anxiety disorder: 32% reported generalized anxiety disorder, 15% reported social anxiety disorder, 16% reported externalizing symptoms, and 13% reported ADHD. Regarding age, Tourette’s is mostly reported at an early age, while mood and psychotic disorders are more frequent in late adolescence [1].

## The role of family in the development and maintenance of pediatric OCD

In pediatric OCD, family plays an important role in the development and maintenance of the disease. Above all, the literature reports evidence on the genetic component of OCD [38]. However, the mode of transmission is unclear, and there is speculation that OCD is a heterogeneous, polygenic, neuropsychiatric disorder [43]. Several studies have identified the possible contribution of a gene (SLCL1A1) on chromosome 9 influencing the glutamate uptake transporter [6, 11]. Additionally, a study by Waters and Barrett [61] examined the role of family characteristics on OCD in children and adolescents. The results demonstrated that 17% of the parents met OCD criteria, with fathers (25%) demonstrating OCD at almost three times the rate of mothers (9%). Also, 13% of parents reported OC threshold symptoms and 20% of fathers exhibited obsessive-compulsive personality traits, compared to 2% of mothers. Regarding siblings, 5% reported OCD, while 35% were considered at risk of developing OCD.

The role of the family in the development and maintenance of OCD in children and adolescents is related to not only genetic factors, but also behavioral factors, including parental modeling and family accommodation. Parental modeling concerns the daily enactment of dysfunctional behavioral patterns by a parent with OCD, which might influence children. For example, an OCD parent’s purification rituals with food could be perceived as normal by their children, who might implicitly learn that food should be cleaned several times before eating due to contamination thoughts [61]. Family accommodation describes the direct participation of parents in their child’s compulsive rituals. For instance, parents might perform rituals on behalf of their child (e.g., checking, cleaning), modify family routines, provide reassurance, or facilitating avoidance of OCD triggers, in order to decrease their child’s distress and time spent executing compulsions [1, 24, 62]. However, parental efforts to relieve their child’s anxiety may inadvertently accommodate and reinforce OC behaviors, and thereby prevent the child from habituating to anxiety and learning that the consequences that are feared typically do not occur. In other words, family members who participate in their OCD child’s rituals might reinforce the child’s belief that it is important to respond to OCD implicit thoughts. In this way, the children may continue to act out OCD-related compulsions but, due to family accommodation, they may not recognize a significant decrease in functioning, as they experience less distress and impairment [9, 55]. At the same time, general family functioning may also decrease, with an increased members’ distress and high levels of family conflict [4, 13, 16]. According to Albert et al. [2] and Wu et al. [62], 80–90% of the relatives of OCD patients directly participate in patients’ rituals linked to symptomatology. High levels of family accommodation are associated with more severe OCD symptom severity, increased internalizing and externalizing symptoms, reduced response to treatment, and greater risk of therapy dropout [20, 56, 62]. Based on this, the American Academy of Child and Adolescent Psychiatry (AACAP), in the practice parameter for the assessment and treatment of children and adolescents with OCD, proposed that: “the role of individual family members in the maintenance and management of OC symptoms is important to assess. For example, detailed and specific questions about activities of daily living may be needed to understand the cycle of OC behaviors at home” [1].

## Family-based treatment in pediatric OCD

The National Institute for Health and Care Excellence (NICE) proposed clinical guidelines for the “Treatment of obsessive-compulsive disorder and body dysmorphic disorder in children and adolescents” [35]. These guidelines indicate that guided self-help interventions, in conjunction with family support, might be particularly effective for OCD children and adolescents with mild functional impairment. However, OCD children and adolescents with moderate to severe functional impairment should be treated with cognitive-behavioral therapy (CBT), integrating exposure and response prevention (ERP) [35]. ERP involves prolonged and repeated exposure to obsessional stimuli without acting out compulsions; this is thought to decrease distress and the perceived necessity to respond to triggering stimuli [29]. The effectiveness of ERP has been demonstrated in OCD children [30] and has been shown to be more effective than pharmacological monotherapy [49] and active psychotherapy (e.g., relaxation therapy) [15, 42]. Currently, CBT with ERP as the core component is the most established and effective psychological treatment for pediatric OCD [14, 15, 37, 44, 59].

The standard treatment for very young children with OCD is cognitive-behavioral family-based treatment (CBFT), which includes the same core components as CBT (including ERP and complementary techniques, such as psychoeducation, cognitive training, and relapse prevention), but with significant family involvement and less focus on cognitive therapy [1, 15]. Parental involvement in the treatment of children with OCD, especially young children, is particularly important, for several reasons. First, poor functioning and high levels of distress, conflict, and blame have been observed in the relatives of children with OCD [29, 39]. Second, family accommodation of OCD symptoms predicts poor treatment response [7, 32, 41]. Finally, parents desire to be engaged in their child’s intervention and to learn how to help their child cope more effectively [47, 54]. In a meta-analysis on the effectiveness of pediatric OCD treatment, higher levels of parental involvement in treatment (i.e., parents attending all treatment sessions and receiving training to assist with ERP) predicted better results than CBT treatment with limited family involvement [46].

Regarding the type of parental involvement, Rosa-Alcazar et al. [45] compared two treatment conditions: CBFT for early-onset OCD, involving both parents and children; and parent training (PT), involving only parents. The results demonstrated that both interventions presented the same efficacy at post-treatment and follow-up in reducing the obsessive-compulsive symptoms of OCD children and adolescents. In other words, the treatment that modified family behaviors through parent training was equally effective as CBFT treatment involving the OCD children/adolescents and their family members [45]. According to our clinical experience, CBFT is preferred in early-onset pediatric OCD, while OCD adolescents could be more amenable to receiving individual CBT, with their parents receiving separate parent training. Overall, these findings show clearly that, in pediatric OCD, family variables play a significant role in patients’ response to CBT treatment. In our opinion, these variables also have a great impact on parents’ level of compliance at each stage of the structured CBT paradigm (e.g., ERP). However, to date, no studies have aimed at delineating practical guidance for psychotherapists to support parents in reducing family accommodation behaviors.

In the following section, we propose a set of practical guidelines to inform the development of a psychoeducational intervention for parents as a core component of any structured CBT program for treating OCD (CBFT or parent training). We propose that, if parents understand the rationale of the CBT treatment and the importance of complying with the exposure instructions, and if they have practical guidance for managing their child’s compulsive rituals at home, their child could benefit from reduced treatment drop-out, post-treatment family accommodation, and OCD symptom severity, and improve their attitude towards ritual to facilitate fewer dysfunctional behaviors.

## Psychoeducational intervention for pediatric OCD

As proposed by Skarphedinsson and Weidle [50], the aim of psychoeducational interventions for parents is to build a common model for understanding the symptoms and treatment of OCD, allowing all parties to work together as a team. The following is a set of basic principles to guide a psychoeducation intervention for the parents of children and adolescents with OCD.

On average, parents receive 12 sessions of treatment. Sessions should be occurred on a weekly basis and should be conducted by one qualified CBT psychotherapist.

In session 1–2, the intervention should seek to build a therapeutic relationship between the psychotherapist and the parents. One strategy to initiate such a relationship would be for the psychotherapist to investigate and inform the parents of their child’s particular strengths. In doing so, the psychotherapist would help parents form a more positive representation of their child, centered on child’s skills and psychological resources; this may contribute to a relaxed atmosphere. Immediately after building a therapeutic relationship, in session 3–5 the psychotherapist should provide parents with basic information about OCD, including the cause of the disease, the prevalence in childhood and adolescence, examples of possible manifestations, the symptomatology, and therapeutic options. There are two fundamental aims of this stage: first, to investigate and modify parents’ possible misconceptions of OCD (e.g., “OCD is just a bad habit or a sign that my child is becoming crazy”); and second, to provide information that accords with the family’s conversational style (e.g., in lay language). Additionally, the psychotherapist should communicate hope, optimism, and an expectation that the OCD symptoms will reduce as a result of the treatment. Finally, to ensure that the information is adequately received by parents, the most relevant information should be repeated (e.g., via a take-home pamphlet), applied to different situations, and illustrated from different perspectives. In this stage, it is important that the parents participate in recognizing the manifestations of the OCD disorder and attributing the symptoms to the disorder, itself, and not to the child. It is essential that parents do not blame their child at this stage, as this would increase their child’s sense of ineffectiveness in dealing with obsessive thoughts and compulsive rituals; this might result in the emergence of depressive symptoms, which could decrease the child’s level of compliance with OCD treatment.

After providing information about OCD and its manifestations, *in session 6–10* psychotherapists should help parents recognize their involvement in their child’s symptoms. Here, psychotherapists may make explicit reference to the concept of family accommodation by explaining to parents how this behavior plays a fundamental role in maintaining the disorder. Therefore, referring directly to the rituals that the child or adolescent patient performs daily, psychotherapists should explain that, although parents may engage in these behaviors to attenuate their child’s OCD-related distress and diminish the time spent performing rituals, doing so is likely to only reinforce their child’s belief that it is important to respond to their OCD implicit thoughts. In this way, the child or adolescent may continue to act out OCD-related compulsions, but, due to the family accommodation, fail to recognize any significant decrease in functioning, as they will experience less distress and impairment.

In this stage of the intervention, the aim is to reduce parents’ criticism and hostile attitudes towards their child by encouraging them to believe that, by adopting more appropriate behaviors, they can contribute to reducing their child’s OCD symptoms. The psychotherapist should share useful behaviors with the parents that can be applied in everyday life to manage their child’s OCD symptoms more effectively in the family’s daily routines. Parents should learn how to avoid getting involved in their child’s compulsive rituals and to facilitate a critical attitude towards the child with respect to these behaviors. At this stage, parent’s intervention into the rituals is not recommended; rather, parents should seek to maintain familiar routines to the greatest extent possible. For example, using role play techniques, the psychotherapist could show parents how to react to a specific compulsive ritual manifested by the child. For example, parents may be encouraged to manage their child’s requests for “reassurance” by explaining that it is not necessary to give reassurance, because it is only the ritual, itself, that is asking for this. Psychotherapists may provide guidance on new behaviors to reduce parental accommodation in the form of homework; in this way, the therapeutic setting can be used by parents to discuss any difficulties they have encountered while attempting to reduce their involvement in their child’s compulsive rituals. Finally, the ultimate and fundamental component of the psychoeducation intervention, in session 11–12, should be to prepare and teach parents the ERP treatment that their child will undergo. Parents should understand that the “exposure” component of this therapy means that their child will expose him/herself to feared stimuli, situations, or thoughts. Furthermore, the “response prevention” aspect means that their child will not perform the rituals or compulsions that are typically associated with the feared stimuli. ERP can be done as defined exercises (e.g., asking the child to touch a “dirty” door handle without washing their hands afterward). It is necessary for parents to be adequately informed on this procedure: indeed, it is fundamental, for the efficacy of the intervention, that they follow the psychotherapist’s indications to ensure that they encourage their child to expose him/herself to feared stimuli. This will prevent them from replacing their child in compulsive rituals and enable them to manage moments of crisis when the child must learn to remain in the frightening situation for a sufficiently long time while not performing the rituals. Consequently, the child should experience that his anxiety either diminishes or becomes tolerable even if he does not perform the rituals. Overall, for the entire duration of the child’s treatment, parents must create a positive atmosphere by obtaining child agreement, maintaining simple but essential communication, recognizing improvements, and avoiding comparisons of outcomes [8]. Such an intervention should be an integral part of CBT for OCD children and adolescents and their families, as it could contribute to reducing OCD symptoms in the family with long-term positive effects [14, 21, 31, 36].

The stages of this psychoeducation intervention and more details of CBT sessions are summarized in Table 1.

**Table 1:** Psychoeducation intervention for parents of children and adolescents with OCD

Session	Objective	Cognitive-Behavioral Key Strategies
Session 1–2 THERAPEUTIC ALLIANCE	− To build a therapeutic relationship between the psychotherapist and the parents	− Build a relaxed atmosphere − Investigate and inform of child’s strengths − Focused on child’ skills and psychological resources
Session 3–5 EDUCATION	− To investigate and modify parents’ possible misconceptions of OCD	− Give information about: cause of the disease, the prevalence in childhood and adolescence, examples of possible manifestations, the symptomatology, and therapeutic options − Communicate hope, optimism, welcoming and restructuring expectation about the OCD symptoms reduction − Attribute symptoms to the OCD itself and not to the child providing information about how to do not blame the child for symptoms − Provide information according to family’s conversational style, repeat and clarify concepts, applied to different situations, and illustrated from different perspectives
Session 6–10 FAMILY ACCOMMODATION MANAGEMENT	− Help to parents: a. To recognize parent’s involvement in child’s symptomatology b. To manage child’s OCD symptoms more effectively in family’s daily routines	− Explain Family Accommodation mechanism (maintenance of the disorder) − Share useful behaviors to apply in everyday life teaching how to avoid getting involved in compulsive rituals − Use role-play techniques to show parents how to react to a child’s specific compulsive rituals
Session 11–12 ERP TREATMENT EDUCATION	− To prepare and to teach parents the ERP treatment	− Psychoeducation about ERP Treatment − Inform adequately parents on the procedure during the specific exposure − Ensure that the parents will follow the psychotherapist’s indication

Further studies and clinical trials are needed to evaluate the efficacy of this practical intervention on OCD children and adolescents, both on the entire psychoeducational intervention than for the specific part that composed it.

## Data Availability

Not applicable.

## Abbreviation

**OCD**: Obsessive-Compulsive Disorder
**CBT**: Cognitive Behavioural Therapy
**ADHD**: Attention Deficit and Hyperactivity Disorder
**ERP**: Exposure and Response Prevention
**CBFT**: Cognitive-Behavioral Family-based Treatment
